# *Bacillus cereus* Induces Severe Infections in Preterm Neonates: Implication at the Hospital and Human Milk Bank Level

**DOI:** 10.3390/toxins13020123

**Published:** 2021-02-07

**Authors:** Delphine Cormontagne, Virginie Rigourd, Jasmina Vidic, Francesco Rizzotto, Emmanuelle Bille, Nalini Ramarao

**Affiliations:** 1 Micalis Institute, INRAE, AgroParisTech, Université Paris-Saclay, 78350 Jouy-en-Josas, France; delphine.cormontagne@inrae.fr (D.C.); jasmina.vidic@inrae.fr (J.V.); francesco.rizzotto@inrae.fr (F.R.); 2 Région Île-de-France Human Milk Bank, Hôpital Necker-Enfants Malades, Assistance Publique-Hôpitaux de Paris, 75015 Paris, France; virginie.rigourd@aphp.fr; 3 Department of Clinical Microbiology, Necker Enfants-Malades Hospital, AP-HP, 75015 Paris, France; emmanuelle.bille@aphp.fr; 4 INSERM U1151-CNRS UMR 8253, Institut Necker-Enfants Malades, Université de Paris, 75015 Paris, France

**Keywords:** *Bacillus cereus*, human breast milk, preterm neonates

## Abstract

Human breast milk (HBM) is a source of essential nutrients for infants and is particularly recommended for preterm neonates when their own mother’s milk is not available. It provides protection against infections and decreases necrotizing enterocolitis and cardiovascular diseases. Nevertheless, HBM spoilage can occur due to contamination by pathogens, and the risk of a shortage of HBM is very often present. *B. cereus* is the most frequent ubiquitous bacteria responsible for HBM being discarded. It can contaminate HBM at all stages, from its collect point to the storage and delivery. *B. cereus* can induce severe infection in newborns with very low birth weight, with sometimes fatal outcomes. Although the source of contamination is rarely identified, in some cases, HBM was suspected as a potential source. Even if the risk is low, as infection due to *B. cereus* in preterm infants should not be overlooked, human milk banks follow strict procedures to avoid contamination, to accurately identify remaining bacteria following pasteurization and to discard non-compliant milk samples. In this review, we present a literature overview of *B. cereus* infections reported in neonates and the suspected sources of contamination. We highlight the procedures followed by the human milk banks from the collection of the milk to its microbiological characterization in Europe. We also present improved detection and decontamination methods that might help to decrease the risk and to preserve the public’s confidence in this vital biological product for infants whose mothers cannot breastfeed.

## 1. Introduction

Very premature infants cannot be breastfed directly by their mothers, but when possible, will receive their own mother’s milk by enteral nutrition. As an alternative, human milk collected, qualified and pasteurized by human milk banks must be used to enhance nutrition or to complete the mother’s milk production. Human milk contains many biologic factors that improve the global outcome of preterm neonates, and formula-fed infants are reported to have 6 to 20 times the risk of experiencing necrotizing enterocolitis compared with breast milk-fed infants. However, despite the obvious beneficial role of human milk, it could also contain bacteria and viruses originating from the milk itself, or from the environment of collection, transport, storage, treatment and administration. Because of the fragility and the high susceptibility of these newborns to infections (prematurity and/or pathology), one of the questions of interest for health professionals is to reduce the risk of bacterial and viral contamination [1]. For a mother’s own raw milk, this question is a direct concern for the neonatal intensive care units. For pasteurized human milk, a human milk bank is the first actor for preventive actions with two aims: (i) prevent milk contamination to avoid preterm infection, and (ii) control the milk process to limit bacteriological rejection and prevent milk shortage [2,3]. During these processes, the main concern is milk contamination by the bacteria *Bacillus cereus*. Indeed, *B. cereus* is the most frequent bacteria found in milk following pasteurization, and it is responsible for a high rate of bacteriologic rejection in human milk banks [4]. Ninety percent of the milk is rejected due to *B. cereus,* which represents 10% of the total volume collected. *B. cereus* is responsible for severe diseases in preterm neonates and the milk has been regularly suspected as the source of contamination [5,6].

The *Bacillus cereus sensu lato* group is composed of seven bacterial species, of which the four best known due to their pathogenicity are *B. cereus sensu stricto*, *B. thuringiensis*, *B. cytotoxicus* and *B. anthracis*. They are Gram-positive, facultative aerobic or anaerobic, sporulating bacteria. These bacteria, which display similar properties, have specific toxins which allow them to colonize hosts as diverse as insects and mammals. *B. cereus* is a saprophytic bacterium, which can be found in a large number of environments, in particular at all stages of the food production chain in hospitals and in human milk banks. The survival of these bacteria along the lines is explained by their ability to produce spores that are resistant to high temperatures, but also able to firmly adhere to materials such as polymers or stainless steels [7]. The presence of bacteria in food is also due to their ability to multiply at low temperatures. *B. cereus* is dangerous from 10^5^–10^6^ bacteria ingested [8]. When ingested, *B. cereus* can cause gastrointestinal infections of varying severity, from mild diarrhea to death due to liver failure or other complications of those infected. *B. cereus* is thus placed in second place of the agents responsible for collective food borne poisoning (FBO) in France and third in Europe [9].

In addition, *B. cereus* is also associated with rare but also more serious non-gastrointestinal pathologies, such as eye infections, pneumonia or meningitis, with sometimes fatal outcomes [10]. In addition, a strain of *B. cereus* carrying a plasmid similar to pXO1 and a plasmid carrying capsule genes, which are known as factors specific to *B. anthracis*, has been responsible for severe anthrax-like infections [11]. *B. cereus* can induce severe pathologies especially in vulnerable children, such as septicemia, respiratory tract infection, enterocolitis, hepatitis, endocarditis, endophthalmitis, encephalitis with cerebral abscess [12,13,14,15,16].

Case reports often focus on individual cases from one hospital only. The overall incidence of local and systemic infections by *B. cereus* is unknown and the characterization of the strains is scarce. A recent study analyzed a survey of infection cases by *B. cereus* from nine different hospitals and proposed a scheme based on biochemical and genetic properties [17]. This revealed that *B. cereus* can be maintained in the hospital environment for up to two years, despite cleaning procedures, and can contaminate unrelated patients, promoting uncontrolled nosocomial infections. Furthermore, in 38% of the cases studied, *B. cereus* was discarded as a simple contaminant. This underlines the fact that not every case of infection is reported and that *B. cereus* incidence is likely underestimated.

Nevertheless, premature infant infection due to *B. cereus* is extremely rare, but neonatal sepsis related to this bacterium remains particularly severe and can be fatal [18,19,20].

## 2. *B. cereus* Induces Severe Pathologies in Preterm Neonates

Cases of infections due to *B. cereus* have previously been reported in immunosuppressed patients. In particular, premature infants and Very Low Birth Weight Infant neonates are highly susceptible to infections because of their immature immune systems and their prolonged exposure to invasive procedures, such as mechanical ventilation and the frequent use of intravascular catheters. However, the pathophysiology of these infections remains poorly understood, with a relatively small number of published cases. Table 1 resumes different clinical forms of *B. cereus* infections reported in the literature. Table 2 relates 42 cases of post-natal infection of *B. cereus* that occurred since December 2016 in Île-de-France (IDF) with the percentages of severe forms and favorable cases. In most of the cases reported in Table 1, the neonates were premature, born before 30 weeks of gestation and, by their young age, had a very low weight: the mean birth was only 27 gestational weeks and the mean baby weight was 1344 g. The sex of the patient was not reported in 76% of the cases and an assessment of a correlation between the sex of the child and infection by *B. cereus* is not possible. Except for the prematurity, a common trait shared by 82% of the cases is low weight at birth (57% for the neonates and 70% for the premature infants, although 24% of the premature cases did not disclose the weight at birth). The patients did not have any other underlying disorders in common.

The preterm neonates were often born without any sign of infection. Nonetheless, because of their premature state, their immune system is not yet mature and they are particularly prone to infection. Most infections developed during the first 10 days of life and the state of the patient quickly declined following the first signs of infection.

Infection was revealed by septic syndromes with positive blood culture (60% of cases) and more or less severe symptomatology, ranging from near-asymptomatic cases to respiratory pauses and cases with extensive and irreversible brain lesions with abscess. Many symptoms were observed, such as respiratory distress with the neonate doing apnea or not breathing by themselves, cardiologic distress with tachycardia and bradycardia, hypotension, increase in leukocytes, which is to be expected during an infection, abdominal distension and, in the worst cases, septic shock, meningitis or cerebral edemas. When sepsis occurred, the mortality was high and frequently led to spontaneous death or medical decision of palliative demarches. In 40% of the cases, an infection by *B. cereus* resulted in the death of the neonate. Histological examination of tissues collected at autopsies of fatal cases of neonatal *B. cereus* infection have demonstrated that tissue invasion with the multiplication of organisms in various organs can also occur [10].

Luckily, *B. cereus* can also cause asymptomatic infections. In the cases identified since 2016 in Île-de-France (Table 2), many patients did not present any symptoms, even though *B. cereus* was found in their blood.

As a whole, newborn neonate infection by *B. cereus* can develop into a multitude of diverse symptoms, which makes it complicated to easily differentiate from other bacteria inducing similar symptoms. The symptoms may indeed be attributed to other bacteria such as *Streptococcus* spp., *Escherichia coli* and *Listeria monocytogenes*, responsible for early bacterial infection disease, or *Staphylococcus aureus* and *Clostridioides* spp., responsible for secondary infectious diseases. In addition, as *B. cereus* was long considered a hospital environment bacterium, a proper diagnosis is sometimes missing or done too late.

Usually, as soon as the symptoms appear, the preterm neonates start an antibiotic treatment, mostly with a combination of antibiotics. Most *B. cereus* are resistant to penicillin and cephalosporin, but they are reported to be susceptible to aminoglycosides, clindamycin, vancomycin, carbapanems, chloramphenicol and erythromycin. Combination therapy with vancomycin and aminoglycoside given empirically has been recommended for systemic infection while awaiting susceptibility data. The efficacy of a treatment of 6 to 8 weeks with an association of vancomycin and carbapenem or ciprofloxacin has also been shown in case of cerebral abscess [17]. Unfortunately, when the first symptoms appear before the eighth day of life, the usual first treatment is ampicillin and/or cephalosporin, which is not efficient against *B. cereus*. Since the infection by *B. cereus* may be rapidly deadly, early recognition of the organism is key for the accurate treatment of the neonates. The pathology of *B. cereus* is serious and needs to be considered more often in predisposed patients. When Gram-positive bacilli are found in blood culture or in the cerebrospinal fluid (CSF), the risks of septicemia or meningitis cannot be overlooked, and an infection by *B. cereus* needs to be considered, especially in preterm neonates.

## 3. Toxins Potentially Involved in *B. cereus* Infection

Clinical manifestations of *B. cereus* have been ascribed to the production of various exotoxins, including the enterotoxin responsible for food borne disease, lecithinase, phospholipase, protease and hemolysins that produce extensive damage and liquefactive necrosis of infected tissues [32].

Diarrheal syndrome is caused by toxins synthesized directly in the intestine by vegetative bacteria. The bacteria are ingested in dairy products, mashed vegetables, or meat dishes, presumably most often in the form of spores [33], which reach the intestine where they germinate, multiply, and produce enterotoxins. The enterotoxins destroy the intestinal barrier, thus causing diarrhea. The contamination is therefore revealed several hours after ingestion, and is manifested by abdominal pain and profuse diarrhea. Currently, three enterotoxins, haemolysin BL (Hbl), non haemolytic enterotoxin (Nhe) and cytotoxin K (CytK), have been described and may play a role in diarrheal symptoms.

Hbl and Nhe are both tripartite toxins. Hbl has three components, the proteins L1, L2 and B. This toxin induces an accumulation of fluid in rabbit ileal loops [34], dermonecrotic activity, vascular permeability [35], cytotoxic activity towards Vero cells and retinal tissues [36,37] and haemolytic activity towards erythrocytes from several species [38,39].

Nhe is a three-protein complex, NheA, NheB and NheC, encoded by the *nhe*ABC operon [40]. This toxin was first characterized in 1995 during a food poisoning outbreak in Norway [41]. Nhe causes cell death through colloid osmotic lysis by provoking a disruption of the plasma membrane and inducing pores in planar lipid bilayers [42]. The *nhe* operon has been found in every *B. cereus* strain so far.

CytK is a single component toxin which is part of the β-barrel pore-forming toxin family. It has cytotoxic and haemolytic activities and demonstrates a similar cytotoxic potency towards cell cultures as Hbl and Nhe [43]. There are two variants of the protein (CytK1 and CytK2), the first one being more toxic than the second [44,45].

The measurement of the level of production of the two enterotoxins Nhe and Hbl in the laboratory environment by immunological kits provides an indication of the pathogenicity of the isolated strains. However, it is not possible to determine a threshold of expression of these enterotoxins discriminating pathogenic strains from non-pathogenic strains. At present, the investigation and epidemiology of toxi-infections with *B. cereus* is difficult due to the lack of a known marker of pathogenesis. Regulatory tests only allow the detection and enumeration of bacteria in suspected foods.

Emetic syndrome results from poisoning; the cereulide toxin is synthesized by *B. cereus* present in food, and quickly (between 30 min and 6 h after ingestion) causes nausea and vomiting. The dishes that cause emetic syndrome are usually based on floury foods such as rice and pasta, prepared in advance, kept at room temperature or poorly refrigerated, and then quickly reheated before tasting. *B. cereus* spores, still present in the food after its initial preparation, germinate during storage and produce the emetic toxin [46]. This toxin is very heat resistant and therefore is not destroyed when reheating food. It is also not broken down by the acidic pH of the stomach or by digestive enzymes, so it can reach the intestine intact. The dose of cereulide sufficient to induce emetic symptoms is estimated to be in the range of 5 to 10 μg/kg body weight, according to tests in monkeys and after analysis of contaminated food. Such a quantity of cereulide can be found in food when a strain of *B. cereus* reaches a concentration greater than or equal to 10^6^ CFU/g. However, no dose-response curve has been established. The strains of *B. cereus* capable of producing the emetic toxin represent a minority (less than 1%) of isolates obtained from food or the environment, but represent 15% of the strains isolated from food that have caused gastrointestinal infections.

In the case of emetic syndrome, it may be necessary to test for cereulide in the offending food, especially if no bacteria are recovered from the food. The detection of cereulide requires the implementation of cell cytotoxicity tests followed by confirmation by mass spectrometry [47]. Cereulide is very stable and can remain in the food after inactivation of the bacteria, for example, by heat treatment. The number of *B. cereus* in food at the stage of its consumption is therefore not a sufficient indicator of the risk of poisoning.

In addition, *B. cereus* produces several other proteins that are toxic *in vitro* or on animal models, which could also contribute to pathologies. For example, the HlyII toxin allows bacteria to resist the host’s immune system by inducing death by apoptosis of macrophages [48,49,50,51]. The proteases InhA1 and NprA allow *B. cereus* spores to escape macrophages after phagocytosis [52,53]. The Mfd protein repairs damage to bacterial DNA caused by nitrogen stress during the host’s immune response to infection [54,55]. CwpFM is involved in the adhesion to epithelial cells and biofilm formation, and CwpFM from pathogenic strains possess structural specificities [56,57].

The role of all these toxins in newborn neonate infections has not been described. The potential roles of these toxins have been studied in vitro and in animal models, but they cannot be considered alone as markers of pathogenicity, and the virulence potential of a strain is likely multifactorial. In addition, as the contamination routes of the newborns are usually unknown, it is difficult to speculate on the impact of one specific toxin on the outcome of the disease.

## 4. Contamination Sources

The sources of patient contamination by *B. cereus* are often suspected but not confirmed. In the case of clinical non-gastrointestinal pathologies, the bacteria can be found in the catheter, in a blood culture or in a wound. Sources of *Bacillus* are not always evaluated, and in less than half of the cases, an environmental or material origin is confirmed (incubator/bed, ventilator equipment, lipids, layers, parenteral solute) [10]. After investigation, the main suspected sources of contamination are the ventilation system, the catheters, but also the feeding tubes, the linens or the benches. Since *B. cereus* is ubiquitous in the environment, the investigations often lead to the discovery of several possible sources of contamination for the patients. *B. cereus* or its spores are even present on collection material considered sterile (in particular, on the wooden stems of swabs) or in antiseptic solutions (alcohol at 95 °C). The isolation of a strain of *B. cereus* from an infection therefore needs to be critically interpreted. Its responsibility for an infection can only be established insofar as *B. cereus* is isolated, in pure culture or in large numbers, several times in the same individual. Furthermore, the strains of *B. cereus* found in the patient’s environment are often different from the isolate of the patient.

In the past few years, advances in neonate care have made the survival of low weight preterm babies possible thanks to invasive equipment. However, this equipment can become a source of infection. The use of mechanical ventilation systems or intravascular catheters during a long period of time make the risks of infections by environmental organisms jump for a vulnerable population. For instance, in 1998, an outbreak of *B. cereus* infection occurred in Amsterdam. Three neonates developed a serious infection, while 35 others were colonized [20]. Approximately one-third of the neonates became infected during their stay. The source of contamination was identified when the interior of balloons used for manual ventilation were positive for *B. cereus*. The isolates from the balloons were the same as the ones infecting the neonates. It is possible that the patients were infected by a direct inoculation of *B. cereus* in the respiratory tract while being manually ventilated. Even though the balloons are often cleaned, the interior is not reached. Furthermore, *B. cereus* has been shown to survive when exposed to cleaning products such as ethanol [58].

**Figure 1:**
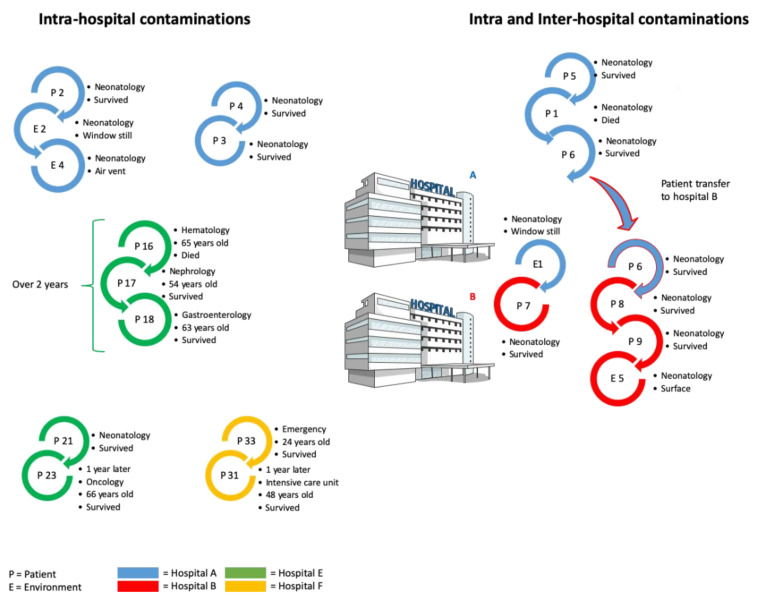
Nosocomial *B. cereus* infections (adapted from [17]).

The hospital environment as a potential source of *B. cereus* contamination has been reported in many studies. However, the fact that *B. cereus* remaining in the hospital environment can infect unrelated patients as a consequence of nosocomial infections was only recently proven [17]. We indeed performed an extensive study of *B. cereus* strains isolated from patients and hospital environments from nine hospitals during a 5-year study, giving an overview of the consequences, sources and pathogenic patterns of *B. cereus* clinical infections. We demonstrated the occurrence of several hospital-cross-contaminations. Identical *B. cereus* strains were recovered from different patients and hospital environments for up to 2 years. We also clearly revealed the occurrence of inter hospital contaminations by the same strain (Figure 1). These cases represent the first documented events of nosocomial epidemy by *B. cereus* responsible for intra and inter hospital contaminations. The contamination of different patients with the same strain of *B. cereus* was so far never shown. However, for each single case, the actual source of infection remains unknown.

Foodborne outbreaks caused by *B. cereus* in neonates are mainly suspected to occur by the ingestion of contaminated dairy products [59,60,61,62]. For instance, *B. cereus* was detected in 27% of pasteurized milk samples collected from major cities in China [59]. Although it is still not directly demonstrated, milk contamination may occur through soil and air that contain a high concentration of *B. cereus* spores: soil contains 50–380,000 CFU/g and air contains <100 CFU/m^3^ of *B. cereus* spores. The production of powered infant formula and the preservation of human milk involve procedures such as pasteurization, which efficiently eliminate vegetative cells of pathogenic bacteria, but not *B. cereus* spores that are resistant to heat, dryness and disinfectants [63]. Besides, the environments for milk production, handling and processing could introduce *B. cereus* into milk. Some studies reported that the storage temperature of dairy products also affected the number and toxicity of *B. cereus*, as toxic strains could produce toxins even at +8 °C [64].

The concomitant occurrence of several cases, and the common nutrition practices with human breast milk (HBM), pointed to the supplied pasteurized human milk as a possible source of contamination. As a first example, we reported a cluster of severe intestinal infections due to *B. cereus* in two very low birth weight neonates. Pooled breast milk was suspected as a source of contamination. However, the strains isolated from the neonates differed from the strains isolated in the milk and in the hospital environment [5]. As another example, in 2016, HBM was suspected as a possible source of *B. cereus* infection in three premature neonates admitted in intensive care units in two hospitals in Île-de-France [21]. Milk batch recall procedure was launched and banked milk production was stopped during the investigation. Five hundred liters of HBM were discarded in regard to commonly used guidelines. The putative role of HBM was raised and microbiological investigation was performed on batches that were used to feed the infants. Despite the absence of bacteria with standard post-pasteurization bacteriological testing, a *B. cereus* isolate was found in an implicated batch upon large sample culture (i.e., 25 mL sampling) and 16 s RNA sequencing for bacterial confirmation. However, after thorough microbiological investigations on retained batches of HBM, no molecular epidemiological relationship and causality could be established between the ingestion of contaminated HBM and neonatal infections, as strains found in HBM and in infected neonates were distinct [21]. Numerous preterm infants have received milk from the same concerned batches without any infectious disease. Finally, additional investigations failed to identify the source of infection. Despite the absence of proven causality, one cannot rule out that the strains that remain on the milk following pasteurization might be dangerous. Thus, *Bacillus* detection and milk controls were reinforced in the two biggest human milk banks of France: the milk bank of Île-de-France and the milk bank of Marmande.

## 5. Food Safety Regulation

*B. cereus* is not subject to food safety criteria according to European regulations. However, since infants are a high-risk category, the products intended for their consumption are subjected to particularly restrictive safety criteria. The amended Regulation No. 2073/2005 defines a process hygiene criterion applicable to *B. cereus* in the case of dehydrated preparations intended for children under 6 months. EU legislation requires the systematic screening of powered infant formula for *B. cereus* contamination (Commission regulation (EC) No 1441/2007). The safety limit for *B. cereus* in foods for children under age of 6 months is established to be 50 CFU/g. Globally, the maximum acceptable number of *B. cereus* is 10^2^ CFU/mL according to the Codex Alimentarius Commission of the Food and Agriculture Organization of the United Nations (FAO) and the World Health Organization (WHO). Such low values are fixed because the *B. cereus* infective dose is 10^3^ CFU/g of food.

In addition, the NF EN ISO 7932 and 21,871 standards allow the identification and enumeration of presumptive *B. cereus* which can be revived.

Especially powdered infant formula, as an effective breast milk substitute, causes a potential safety risk to newborns and infants’ health because it is not a sterile product. In 2014, Brandl et al. [65] showed the presence of about fifty different aerobic culturable microorganisms in the air of Swiss milk processing facilities. Among them, *B. cereus* was one of the most frequent and most harmful bacterial groups affecting the safety of powdered milk. Their spores strongly resist stress conditions encountered in the production of powdered products. The detection of spores is an even longer procedure than that for vegetative cell detection because spores firstly need to germinate, and only formed vegetative cells are detected using standard procedure for *B. cereus* bacteria. In addition, spores may germinate directly in milk, forming vegetative cells productive of toxins or spoilage enzymes [66]. Currently, the genes *hbl*, *nhe*, *cwpFM, cytK* and *hlyII* encoding toxins are targeted in powdered milk by PCR, RT-PCR or multiplex-PCR [59].

Emetic strains have been isolated from raw milk, and the isolated emetic toxin, cereulide, showed high toxicity, highlighting the importance of detecting and eliminating emetic toxins and strains from raw milk [67]. Progress has been made in the detection of the emetic toxin, cereulide [68]. These methods include mass spectrometry, allowing for cereulide detection in milk [69]. In addition, emetic *B. cereus* strains possess specific growth characteristics, and this should be taken into account to prevent the risk of emetic food poisoning [70].

The large number of potential toxin genes related to *B. cereus* virulence strongly limits the efficiency of existing techniques and official diagnostic methods for *B. cereus*, and virulence factors still have to be developed. Similarly, an efficient method for the direct detection of *B. cereus* spores in milk and infant formula (without the need for a germination step) is still missing. We have recently reviewed analytical methods under development for *B. cereus* spores and toxin detection [60,71]. The time needed for their validation and subsequent adaptation by hospitals is uncertain.

## 6. Human Milk Banks—Methods, Practices and Issues

The Île-de-France milk bank collects, pasteurizes, qualifies and distributes approximately 12,000 L of human milk necessary to feed approximately 3500 preterm neonates annually in complement to their own mother’s milk. Production procedures, such as collection, preparation and post-pasteurization microbiological analyses for human milk bank qualification, follow ADLF’s (Association Française Des Lactariums de France (http://association-des-lactariums-de-france.fr)) standards.

### 6.1. General Hygiene Procedure and Milk Treatment

**Figure 2:**
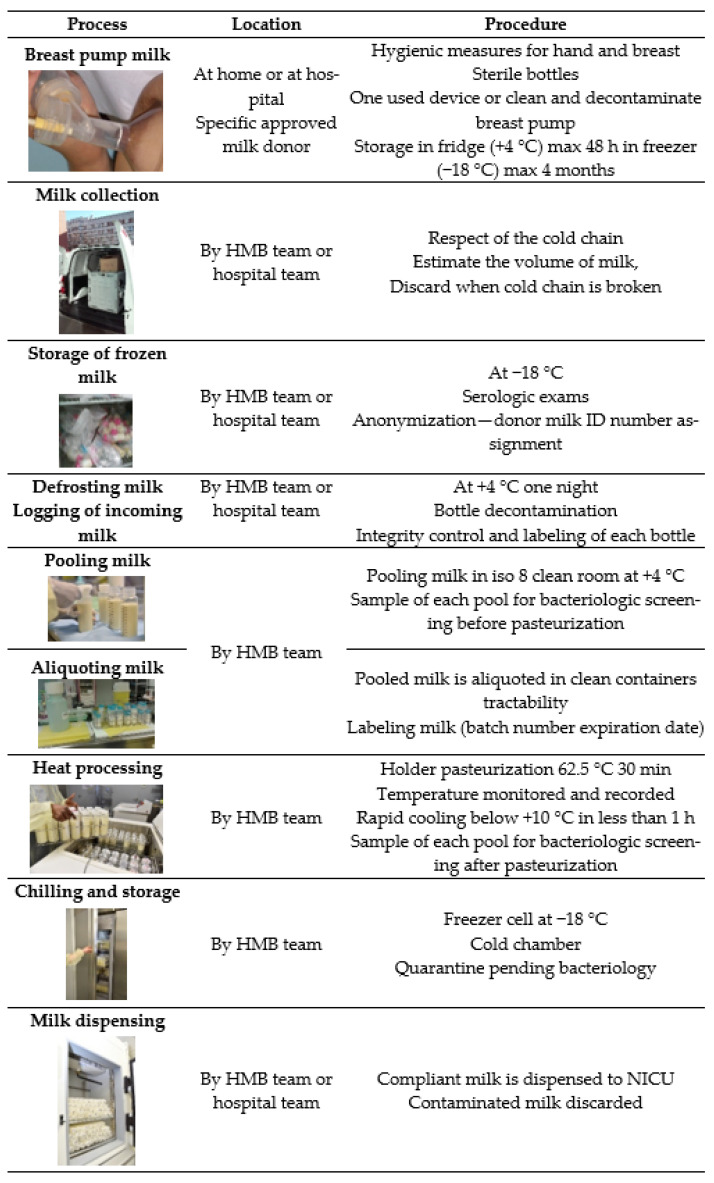
Standard procedure for milk collection and handling. HMB: Human Milk Bank; NICU: Neonatal Intensive Care Unit.

The regulation regarding preventive hygiene practices is specific to each country [2,3] (https://www.edqm.eu/en/organs-tissues-and-cells-technical-guides, Chapter 33). In France, for instance, the reference legislation is the article L. 2323-1 of the public health code (https://www.legifrance.gouv.fr/codes/article_lc/LEGIARTI000025104626/). It defines the quality management and control system to be applied, from staff training to the use of rooms and equipment. The procedures for the collection, control, processing and storage of donated milk are defined as well. All materials used by donors and staff are sterilized and provided by the institution. Any material in contact with the skin or milk is systematically washed and decontaminated. The transport of milk, from collection to its distribution, is carried out in strict compliance with the cold chain. Figure 2 resumes the different steps of human milk treatment from the breast pump to the administration to the preterm neonates.

As a first step, regular cleaning and disinfection of surfaces and material used for breast milk pumps with an ordinary household disinfectant containing a 1% dilute chlorinated solution appears as an essential barrier measure. As recommended by the Centers for Disease Control of Prevention (2020), World Health Organization (2020) and Milk Banking Association (2020), “after each pumping session, all pump part that come into contact with breast milk should be appropriately disinfected”. It was also demonstrated that steam decontamination using a microwavable bag after washing resulted in a lower proportion of discarded HBM (1.3% vs. 18% *p* < 0.001). The French Food Safety Agency recommended sterile single-use or autoclavable sterilized or bacteriological clean breast pump milk collection kits for mothers in hospital. An important step is the bio-cleaning of the pump and its accessories with a detergent/disinfectant followed by boiling in water for 20 min, pressure cooker for 10 min, steam or microwave sterilizers, which constitutes by far the most widespread method of decontamination; or, less common but also less time consuming and probably easier to apply, using chlorine solution (CS) [72]. CS would provide a simple solution to numerous mothers to improve the safety of their maternal milk, and we have recently published that it is safe for the infant [73]. This could especially apply to women living in precarious conditions or women who breast pump their milk at work and do not have access to boiling water for decontamination. The second step is to prevent milk contamination with skin flora. Usually *B. cereus* is not concerned, unlike *Staphylococcus*, and an easy process such as hand and breast washing with soap before pumping breast milk is sufficient.

Strict respect of the cold chain temperature of the milk at all the stages of its management must be guaranteed. This concerns the donor at home, the storage of milk at the human milk bank and its transport during delivery. For transport between 30 min and 2 h, the milk must remain refrigerated (between 0 °C and 8 °C) or frozen (below −10 °C) according to its initial state of preservation. For transport exceeding 2 h, breast milk must be transported frozen and stored at a temperature between −10 °C and −30 °C.

Milk treatments after microbiological analysis usually consist of a pasteurization (at 62.5 °C for 30 min) and a fast refrigeration [74]. Other thermal pasteurization conditions (72–75 °C) and a few non-thermal processes (high pressure processing, microwave irradiation) have also been investigated [75]. In any case, a new microbiological analysis has to be performed and the results must be negative for each lot. Milk is kept in quarantine before being analyzed for microbiological safety. The milk must therefore be frozen and kept in special storage compartments for the entire storage period prior to use. A freeze-drying process can optionally be carried out as an alternative to freezing. Finally, the appearance of the product, the integrity of the container and the labeling must be checked during distribution and dispensing.

### 6.2. B. cereus in HBM

**Figure 3:**
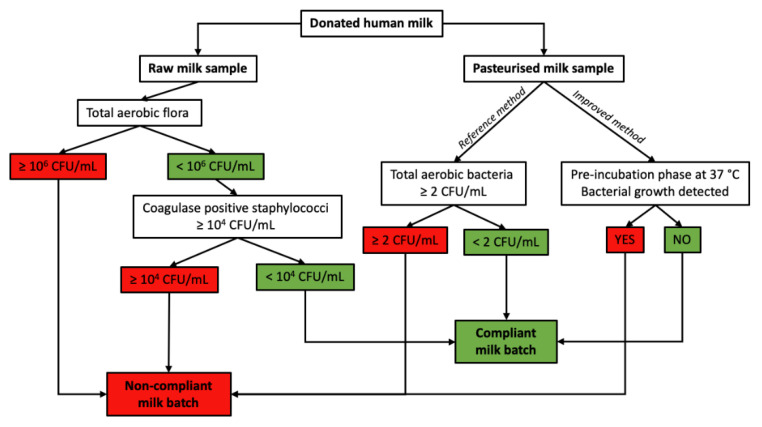
Good practice rules according to paragraph 3 of article L. 2323-1 of the French Public Health with reference and improved method.

At arrival at the human milk bank (HMB), the raw HBM is firstly analyzed by bacteriological analysis. Then, post-pasteurization of the milk, a mandatory second bacteriological analysis, is carried out. Milk banks have established safety standards that define the acceptance or rejection criteria prior to distributing pasteurized milk. For the pre-pasteurized milk, an aerobic total flora count on blood agar and a coagulase-positive *Staphylococci* count on a specific medium after 48 h of incubation at 37 °C have to be performed. Batches shall be declared non-compliant if the aerobic flora is equal to or greater than 10^6^ CFU/mL, or if the number of coagulase-positive *Staphylococci* is equal to or greater than 10^4^ CFU/mL. For a pasteurized milk sample, a 0.5 mL volume is spread onto a rich medium and incubated aerobically at 37 °C for 48 h. Following pasteurization, any batch with quantitative culture ≥2 CFU/mL is destroyed. For *B. cereus*, all currently available milk bank guidelines recommend the release of milk only once cultures are completely negative. A negative result for *Bacillus* in a post-pasteurization culture does not mean that this micro-organism is absent, but only that it is under the detection limit by the technique (e.g., 10^2^ CFU/mL if 10 mL of milk was cultured). To be compliant and used, the HBM should respect the three criteria: total flora below 10^6^ CFU/mL and coagulase-positive *Staphylococci* below 10^4^ CFU/mL before pasteurization, as well as total flora below 2 CFU/mL post pasteurization (Figure 3).

**Figure 4:**
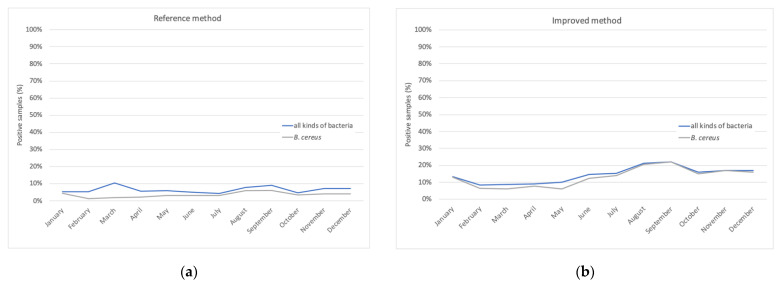
Contaminated milk samples post-pasteurization detected with reference method (**a**) and improved method (**b**).

Following these methods and recommendations, the analysis revealed that, on average, 7 to 25% of the samples are still contaminated following pasteurization treatments (Figure 4a). Interestingly, we can also notice a variation in contamination according to the season. Identification of the bacteria surviving the pasteurization process shows that *B. cereus* and *Streptococcus thermophilus* are almost the unique remaining bacteria (Figure 4a). This is not surprising as *B. cereus* spores have been shown to resist heat treatments up to 70 °C [76].

As said previously, HBM has been suspected in some cases as a potential source of *B. cereus* contamination of preterm neonates, although a causality has never been proven. Nevertheless, as *B. cereus* is a major HBM contaminant surviving the pasteurization process, it is of paramount importance to perform risk assessment of *B. cereus* in pasteurized milk. Consistently, and following the 2016 incidents, the Necker human milk bank has decided to use an even sensitized post-pasteurization analysis for *B. cereus* (Figure 3). On arrival at the laboratory, 20 mL of the sample is incubated for 18 h at 37 °C under aerobic conditions (pre-incubation or enrichment stage). This step encourages any spores present in the milk to enter the vegetative cycle. At the end of the pre-incubation period, 50 μL of milk is used to seed a Petri dish containing Colombia-horse blood medium, incubated for at least 18 h at 37 °C under aerobic conditions. Each type of colony present on the agar plates is then identified by MALDI-TOF mass spectrometry. With this “improved” method, 71% of the positive batches with *B. cereus* were negative with the reference method, but identification of the germs allowed the determination of whether the isolated germs were non-pathogenic and/or known to be destroyed by pasteurization, suggesting a contamination of the sample during analysis, but not a contamination of the milk lot. The improved method thus avoids false positive samples.

Historically (Figure 4a, Table 3), 7% of the milk batches produced by our milk bank have been disqualified because of microbial contamination; half for pre-pasteurization contamination (total flora >10^6^ CFU/mL or coagulase-positive *Staphylococci* >10^4^ CFU/mL) and the other half for post-pasteurization contamination (thermal resistant bacteria or sporulated bacteria). A modification of bacteriologic control of post-pasteurized milk has unsurprisingly increased the rate to 15% and even 21% in 2019 post-pasteurization non-conformity (Table 3). *B. cereus* was the microorganism found in 80–90% of the batches that were disqualified (Figure 3B). Thus, we have developed a bacteriological analysis method more sensitive than the reference method for the research of *B. cereus* in pasteurized woman’s milk delivered by the human milk bank. This analysis also allows us to limit discharges for bacteriological nonconformities due to contamination (when handling the sample or in the laboratory) or the presence of non-pathogenic thermophilic germs, such as *S. thermophilus*.

A Canadian analysis has evaluated the number of cases prevented by an improved method versus the number of discarded liters of milk [31]. They have estimated the potential risk of *B. cereus* infection in preterm infants caused by the ingestion of contaminated pasteurized HBM using different post-pasteurization release criteria (i.e., 9 samplings of 100 microliters versus the human milk bank association of north America (HMBANA) guideline of 1 sampling of 100 μL per pool). A simulation highlights the very small risk of *B. cereus* infection following the ingestion of pasteurized HBM, even in the worst cases scenarios, and suggests that a 100-μL sample for post-pasteurization culture is sufficient to mitigate this risk. A larger sampling volume would only lead to a higher rate of disqualification for this important health-care resource, without having any significant positive impact on safety.

To decrease *B. cereus* milk contamination, a new process of pasteurization was also developed. It was determined that heating for 32 min at 48 °C, 20 min at 50 °C, 4 min at 54 °C, and 0.4 min at 60 °C would be sufficient for a 6 log10 CFU/mL reduction (t-6 log(T)) in vegetative cells of the most heat-resistant among the six psychrotolerant strains tested. These results suggest that the reheating of food products before consumption could rapidly eliminate psychrotolerant *B. cereus* vegetative cells, if storage conditions had permitted their multiplication in the food products. However, reheating would not inactivate cereulide, the heat stable emetic toxin produced by some rare psychrotolerant strains of *B. cereus*, but will also more drastically deprive the milk of its anti-infection properties such as IgA (Secretory Immunoglobuline A), lactoferrin and lysozyme.

Deep freeze is also a way to minimize post-pasteurization contamination by making the hypothesis that thermal shock prevents the multiplication of aerobic spores [2]. Furthermore, deep freezing does not alter the milk nutritional quality [2,77,78]. The acceleration of the freezing procedure certainly has a beneficial role by limiting the time when the milk temperature is between 30 °C and 37 °C and preventing the risk of entry into the vegetative cycle effective between +10 °C and +4 °C.

We conducted a comparative study using the improved detection protocol on post-deep freeze versus post-pasteurization milk. Thirty-four samples were analyzed. Table 4 resumes the results of bacteriology post-pasteurization and post pasteurization-deep freezing. Thirty-two percent of non-compliant milk after pasteurization was compliant after pasteurization/deep freezing. The implementation of this new pasteurization/deep freezing method on these samples, which have become compliant, shows that they correspond to concentrations of less than 0.07 CFU/mL. This interesting approach could be widely used by neonatalogy services. This would reduce losses while pursuing a strengthened method of detecting *B. cereus*, therefore achieving maximum safety for the final product.

Taken together, improved detection and decontamination methods might help decreasing the risk and help to preserve the public’s confidence in this vital biological product for infants whose mothers cannot breastfeed.

## 7. Conclusions

In this review, we have gathered information on various *B. cereus* cases and outcomes in preterm neonates. These data clearly indicate that newborn infections by *B. cereus* should not be overlooked. The range of symptoms is wide and it is difficult to associate a particular disease to *B. cereus*. The infection can be asymptomatic to severe, with brain lesions and septic shock, to lethal. It is key to identify as quickly as possible the agent responsible for the infection in order to pursue a line of treatment adapted to *B. cereus*. As such, *B. cereus* should not be discarded as a contaminant and appropriate antibiotic treatment needs to be given promptly.

The contamination sources of *B. cereus* are often found to be in the hospital equipment—in particular, the ventilation system and the catheters. The spores are highly resistant and can be found in sterile material and even in antiseptic solutions. Even though the strains found in the environment of the patient are often different from the strain infecting them, the identification of the source is often lacking. In a few cases, HBM has been suspected to be the source of contamination, but no correlation between the strain found in the milk and the one infecting the patients could be found. Nonetheless, since HBM could be a potential source of contamination, the detection and the control in the milk are sometimes reinforced and follow a strict protocol. As such, the risk appears extremely low and is mitigated by post-pasteurization microbiological criteria applied to define HBM acceptance for distribution. New technology applied in human milk banks such as high pressure could be a real improvement as it totally destroys *B. cereus* spores. It could reduce discarding human milk while preserving a maximum of anti-infectious properties. Before high pressure becomes available, bacteriology post deep-freezing seems to be a good opportunity to reduce residual *B. cereus* in pasteurized human milk.

Given the widespread dissemination of *B. cereus* in hospital, and the important risks for preterm neonate children, more emphasis should be given to the immediate Intensive Care Unit environment, such as invasive care equipment, linens, ventilation and air conditioning systems.

## Figures and Tables

**Table 1:** *B. cereus* infection in neonates. Literature review (NS: Not specified; ND: Not determined; F: Female; M: Male; ET: Endotracheal tube; CSF: Cerebrospinal fluid; HBM: Human breast milk).

Ref	Sex	Birth Weight (g)	Gestational Age, Weeks + Days	Age at First Positive Culture, Days	Predisposing Factors	Treatment	Outcome	Suspected or Proven Source of Infection	First *B. cereus* Identification	Toxin Identified
[21]	NS	750	30 +2	3	Premature, low weight	NS	Died	Pasteurized milk, packs of diapers, linen, baby bath, benches (suspected)	Blood culture	ND
	NS	3000	40	1		NS	Survived		Cavum	ND
	NS	1075	29 + 2	5	Premature, low weight	NS	Died		Blood culture	ND
	NS	2815	37 + 2	2		NS	Survived		Blood culture	ND
	NS	3515	38 + 6	6		NS	Survived		Blood culture	ND
	NS	3240	39	9		NS	Survived		Blood culture	ND
	NS	1380	31	9	Premature, low weight	NS	Survived		Blood culture	ND
	NS	1025	29 + 4	11	Premature, low weight	NS	Survived		Blood culture	ND
	NS	750	27 + 5	76	Premature, low weight	NS	Died		Blood culture	ND
	NS	1720	31	10	Premature, low weight	NS	Survived		Blood culture	ND
[18]	F	880	27 + 2	4	Premature, low weight	Cefotaxime, gentamicin, vancomycin, fluoroquinolone	Died	Incubator, ultrasonographic probe, bench used for bottle feeding (suspected)	Tracheobronchial aspiration	CytK2, Nhe
	M	1480	29 + 4	4	Premature, low weight	Cefotaxime, gentamicin, vancomycin	Died		Blood culture	
[22]	NS	1650	31	70	Premature, low weight	Vancomycin	Survived	Bioaerosol and surface contamination. Work stations, storage room, linens (suspected)	Blood culture	ND
	NS	1148	29	58	Premature, low weight	Vancomycin	Survived		Blood culture	ND
	NS	1515	28	23	Premature, low weight	Vancomycin	Survived		Blood culture	ND
	NS	710	24	14	Premature, low weight	Vancomycin	Survived		Blood culture	ND
	NS	945	25	59	Premature, low weight	Vancomycin	Survived		Blood culture	ND
[23]	M	1580	26	30	Premature, low weight	Ampicillin, cefotaxime then amikacin and vancomycin	Died	ND	Blood culture	ND
[5]	NS	960	29	3	Premature, low weight	Vancomycin, cefotaxime and metronidazole	Survived	Food-related origin: milk (suspected)	Gastric fluid culture	ND
	NS	1500	30	3	Premature, low weight	Vancomycin, cefotaxime and metronidazole	Survived		Gastric fluid culture	ND
[24]	F	1670	31	28	Premature, low weight	Cefotaxime, amoxicillin, metronidazole, amikacin	Died	ND	Blood culture and central catheter	ND
[25]	NS	880	27	1	Premature, low weight	Vancomycin	Survived	Water from washing machine chamber (proven)	ET aspirates	ND
	NS	880	28	1	Premature, low weight	Vancomycin	Died		ET aspirates	ND
	NS	720	27	3	Premature, low weight	ND	Survived		ET aspirates	ND
	NS	880	29	3	Premature, low weight	ND	Survived		ET aspirates	ND
	NS	640	28	2	Premature, low weight	Vancomycin	Died	Vial of pulmonary surfactant used for both of them (but no growth)	ET aspirates	ND
	NS	530	26	3	Premature, low weight	Vancomycin	Died		ET aspirates	ND
[26]	F	800	NS	1	Premature, low weight	Vancomycin, meropenem and for meningitis: linezolid, meropenem and clindamycin	Survived	Ventilator equipment, intravascular catheters and linen (suspected)	Blood culture	ND
[27]	F	830	26	8	Premature, low weight	Vancomycin, amikacin	Died	ND	Blood culture	ND
[19]	NS	650	24 + 5	32	Premature, low weight	Cefotaxime, vancomycin and amikacin	Survived	Arterial catheter (suspected)	Blood culture	Nhe
	NS	615	26 + 5	5	Premature, low weight	NS	Died	Catheter (suspected)	Peripheral catheter and central catheter	Nhe
[28]	M	NS	NS	1	NS	Cefozopran	Died	Hospital linens (proven)		ND
	F	NS	NS	19	NS	Ampicillin, meropenem, vancomycin, panipenem	Died			ND
[29]	F	3764	37	6	Premature	Ampicillin, gentamicin	Died	Peripheral vein catheter (suspected)	CSF and blood culture	ND
	F	1506	36	9	Premature, low weight	Ampicillin, cefotaxime	Died	Nasal feeding tube (suspected)	CSF culture	ND
[20]	M	895	28 + 5	5	Premature, low weight	Amoxicillin and cefotaxime	Died	Balloons of manual ventilation, person to person transmission (health care workers hands) (suspected)	Blood culture, CSF, trachea aspirate, necrotic Skin lesion at insertion site of arterial catheter	ND
	F	1000	26 + 4	5	Premature, low weight	Meropenem, vancomycin	Survived		Blood, aspirate from left knee	ND
	M	2780	37 + 3	14	Premature	Meropenem, vancomycin	Survived		Blood, CSF, tip of peripheral catheter	ND
[30]	M	585	24	19	Premature, low weight	Vancomycin, tobramycin	Survived	ND	Blood culture	ND
[31]	NS	590	25	10	Premature, low weight	Linezolid, meropenem, vancomycin	Died	Batches of HBM but the strains from the baby are different (suspected)	Blood culture	ND
	NS	560	24	6	Premature, low weight	Ampicillin, tobramycin, vancomycin, meropenem, piperacillin-tazobactam, fluconazole, amphotericin and trimethoprim-sulfamethoxazole	Died	HBM (suspected)	Blood culture	ND
[32]	NS	NS	NS	NS	Premature		Died	ND	Blood culture and cerebrospinal fluid	Nhe
	NS	NS	NS	NS	Premature	Vancomycin, cefotaxime	Survived	ND	Blood culture	Nhe, Hbl
	NS	NS	NS	NS	Premature	Vancomycin	Survived	ND	Blood culture	Nhe
	NS	NS	NS	NS		Vancomycin	Survived	ND	Neonatal gastric liquid	Nhe
	NS	NS	NS	NS		Cefotaxime, amoxicillin, amikacin	Survived	ND	Umbilical	Nhe
	NS	NS	NS	NS		Ceftriaxone	Survived	ND	Axilla later feces	Nhe
	NS	NS	NS	NS	Premature	Cefotaxime, amoxicillin, amikacin	Survived	ND	Stomach tube feeding	Nhe
	NS	NS	NS	NS	Premature	Vancomycin	Survived	ND	Gastric acid	Nhe
	NS	NS	NS	NS	Premature	Amoxicillin, amikacin, vancomycin	Survived	ND	Central venous catheter	Nhe
	NS	NS	NS	NS	Premature	Vancomycin, cefotaxime, amikacin	Survived	ND	Blood culture	Nhe
	NS	NS	NS	NS	Premature		Died	ND	Blood culture	Nhe
	NS	NS	NS	NS	Premature	Vancomycin, cefotaxime, metronidazole	Survived	ND	Stomach tube feeding	ND
	NS	NS	NS	NS	Premature	Vancomycin, cefotaxime, metronidazole	Survived	ND	Stomach tube feeding	ND
	NS	NS	NS	NS		Ceftriaxone, gentamicin	Survived	ND	Blood culture	ND
	NS	NS	NS	NS	Premature	Vancomycin	Died	ND	Blood culture from umbilical venous catheter and peripheral veins	Nhe, Hbl
	NS	NS	NS	NS	Premature	Vancomycin	Died	ND	Bronchial aspiration (lung)	Nhe

**Table 2:** *B. cereus* infection cases reported in Île-de-France since 2016 (WA: Week of amenorrhea; UVC: Umbilical venous catheter; LS: lipids in parenteral solution; MB: milk from Human Milk Bank; OMM: own mother’s milk; HL: artificial milk type hydrolysate).

Term and Birth Weight	Hours or Days After Birth	Bacteriological Analyses	Symptoms	Evolution
25 WA 675 g	D14	Blood (+) UVC(+) LS (+) MB (-)	Diffuse multiple abscess 2 hemispheres	Death
39 WA + 1 2500 g	D22	Blood (+) OMM (-) HL	Respiratory breaks	Favorable
25 WA + 1 665 g	D134	Blood (+) OMM(-)	Respiratory breaks	Favorable
25 WA 600 g	D15	Blood (+) OMM (-)	No symptom	Favorable
38 WA 2700 g	D32	Blood (+) HL	No symptom	Favorable
26 WA 675 g	D26	Blood (+) MB (-)	No symptom	Favorable
ND		Blood (+) Parenteral	No symptom	Favorable
28 WA + 5 585 g	D13	Blood (+) Incubator (+)	No symptom	Favorable
32 WA 1530 g	D3	Blood (+) MB (-)	No symptom	Favorable
ND		Blood (+) MB (-)		Favorable
29 WA + 5 1240 g	D20	Blood (+)	No symptom	Favorable
31 WA 1410 g	D4	Blood (+) MB (-)	No symptom	Favorable
25 WA 635 g	D6	Blood (+) MB (-)	Respiratory breaks	Favorable
29 WA 1260 g	D18	Blood (+)	Respiratory breaks	Favorable
31 WA + 2 1940 g	D16	Blood (+) MB(-)	No symptom	Favorable
25 WA + 6 855 g	D6	Blood (+) Peritonitis MB (-)	Increase needs in 0_2_	Favorable
37 WA + 2 2035 g	D16	Blood (+)	No symptom	Favorable
28 WA + 2 865 g	D16	Blood (+) MB (-)		Favorable
4 premature babies		Blood (+) MB (-)	One with a brain abscess	1 Death/4
1 premature baby		MB (-)		Favorable
1 premature baby		Blood (+) LS (+) MB (-)		Death
29 WA 1210 g	D21	Electric ramp (+) Blood (+ *Bacillus pumilus*) MB (-)	Bone localization	Favorable
29 WA + 5 1285 g	D6	Blood (+) MB (-)	Extensive periventricular hemorrhage and cytotoxic Involvement of the cortex and central gray nuclei	Death
1 premature baby		Blood (+) MB (-)		Favorable
31 WA + 2 1430 g	D29	Blood (+) MB (+ 10^3^ *Bc*)		Favorable
		Blood (+)		Favorable
Near born		Blood (+) Incubator (+)		
25 WA	D19	2 Blood (+) *Bacillus subtilis* MB (-)		Favorable
ND		Tracheal suction (+) MB (-)		
ND		MB (-)		
1 premature baby		Blood (+) MB(-)		
1 premature baby		Blood (+) MB (-)		
29 WA 550 g	D3	Blood (+) Parenteral (+)	Brain abscess	Death
30 WA 740 g	H50	Blood (+)	Brain abscess	Death
36 WA 2000 g	D1	Nasopharynx (+)		Favorable

**Table 3:** Volume of human milk unfit for consumption as % of total volume collected.

Year	Bacteriologic Contamination Liters (%)
2000	192 (3.3%)
2001	174 (3.4%)
2002	172 (3.2%)
2003	155 (2.5%)
2004	256 (4%)
2005	215 (3.3%)
2006	249 (3.4%)
2007	293 (4.2%)
2008 *	524 (8.1%)
2009	401 (6.4%)
2010	465 (7.2%)
2011	541 (8.1%)
2012 **	628 (11.3%)
2013	515 (8.1%)
2014 ***	1221 (14.5%)
2015	815 (9.6%)
2016 ****	1097 (13.9%)
2017 ^■^	1851(19.45%)
2018	2018 (19%)
2019	2463 (21.2%)

* Hardening of bacteriological standards official guidelines. ** Necker transfer. *** Significant raw milk session by other Human Milk Banks with a bacteriological rejection rate of 20%. **** Introduction of the enhanced method following the *B. cereus* crisis. ^■^ Generalization of the strengthening of post-pasteurization bacteriology, sensitization towards *B. cereus*.

**Table 4:** Impact of pasteurization and fast freezing on *B. cereus* contamination.

Date	Culture Post Pasteurization	Culture Post Pasteurization/Fast-Freezing
21-Feb	*Bacillus cereus*	Negative
16-Apr	*Bacillus cereus*	*Bacillus cereus*
28-May	*Bacillus cereus*	Negative
11-Apr	*Bacillus cereus*	*Streptococcus thermophilus*
29-May	*Bacillus cereus*	*Bacillus cereus*
05-Apr	*Bacillus cereus*	*Bacillus cereus*
13-May	*Bacillus cereus*	Negative
29-Mar	*Bacillus cereus*	Negative
10-Apr	*Bacillus cereus*	Negative
05-Apr	*Bacillus cereus*	Negative
07-Mar	*Bacillus cereus*	*Bacillus cereus*
04-Mar	*Bacillus cereus*	Negative
12-Mar	*Bacillus cereus*	*Bacillus cereus*
21-Mar	*Bacillus cereus*	*Bacillus cereus*
18-Mar	*Bacillus cereus*	*Bacillus cereus*
16-Apr	*Bacillus cereus*	Negative
03-Jun	*Bacillus cereus*	*Bacillus cereus*
28-Feb	*Bacillus cereus*	*Enterococcus faecalis*
20-Feb	*Bacillus cereus*	Negative
14-May	*Bacillus cereus*	*Bacillus cereus*
22-May	*Bacillus cereus*	Negative
27-Feb	*Bacillus cereus*	*Bacillus cereus*
27-Feb	*Bacillus cereus*	*Bacillus cereus*
04-Feb	*Bacillus cereus*	Negative
08-Feb	*Bacillus cereus*	*Bacillus cereus*
05-Feb	*Bacillus cereus*	*Bacillus cereus*
15-Feb	*Bacillus cereus*	*Bacillus cereus*
27-Feb	*Bacillus cereus*	*Bacillus cereus*
06-May	*Bacillus cereus*	*Bacillus cereus*
12-Feb	*Bacillus cereus*	*Bacillus cereus*
27-Feb	*Bacillus cereus*	*Bacillus spp*.
07-Feb	*Bacillus cereus*	*Bacillus cereus*
12-Feb	*Bacillus cereus*	*Bacillus cereus*
01-Mar	*Bacillus cereus*	*Bacillus cereus*

## Data Availability

No new data were created or analyzed in this study. Data sharing is not applicable to this article.
